# Transformer-Based Reinforcement Learning for Multi-Robot Autonomous Exploration

**DOI:** 10.3390/s24165083

**Published:** 2024-08-06

**Authors:** Qihong Chen, Rui Wang, Ming Lyu, Jie Zhang

**Affiliations:** School of Automation, Nanjing University of Science and Technology, Nanjing 210094, China; qh_chen_njust@163.com (Q.C.); wangrui_au@njust.edu.cn (R.W.); minglyu_njust@163.com (M.L.)

**Keywords:** deep reinforcement learning, robot exploration, artificial neural network

## Abstract

A map of the environment is the basis for the robot’s navigation. Multi-robot collaborative autonomous exploration allows for rapidly constructing maps of unknown environments, essential for application areas such as search and rescue missions. Traditional autonomous exploration methods are inefficient due to the repetitive exploration problem. For this reason, we propose a multi-robot autonomous exploration method based on the Transformer model. Our multi-agent deep reinforcement learning method includes a multi-agent learning method to effectively improve exploration efficiency. We conducted experiments comparing our proposed method with existing methods in a simulation environment, and the experimental results showed that our proposed method had a good performance and a specific generalization ability.

## 1. Introduction

The environment map serves as the foundation for the robots’ later tasks, such as navigation. Multi-robot autonomous exploration entails employing a group of robots that navigate unknown areas, scan their surroundings, and cooperate to construct a map of the environment [1]. Robotic autonomous exploration is extensively employed in various domains, including manufacturing environment construction [2], sweeping robots [3], search and rescue mission [4], and planetary exploration [5].

Multi-robot autonomous exploration can be classified into two categories, centralized and distributed, in terms of system architecture [6]. In a centralized multi-robot autonomous exploration system, complete information is exchanged among the robots [7]. Conversely, communication is limited in a distributed multi-robot autonomous exploration system [8]. In a centralized system, the exploration approach enables the robot team to be perceived as a unified entity and devise effective plans by fully exchanging information. In a distributed system, since individual robots can only use their local observation information and limited communication with their teammates, they need to infer their teammates’ intentions to avoid repeated scanning of the areas explored by their teammates.

The frontier is an important concept in robotic autonomous exploration, which refers to the boundary between free space and unknown space in the map being constructed [9]. The nearest frontier is a classical approach to multi-robot autonomous exploration. It accomplishes the exploration of the environment by continuously selecting the nearest frontier location as a goal point for each robot. Although this method is uncomplicated, it poses challenges for robot teams to disperse, resulting in extensive redundant exploration and inefficiency. The utility-based approach is another traditional approach [10]. This approach manually constructs a utility function consisting of multiple terms that combine the balance between information gain and loss, such as exploration area, travel distance, and robot dispersion. However, the increase in the number of factors considered can lead to a very complex utility function that is difficult to solve.

Artificial neural network technology has led to using learning-based methods for decision-making tasks [11]. Alpha Go is a Go matchmaking software that utilizes artificial intelligence and has successfully outperformed the most skilled human players [12,13]. Mnih et al. employed deep reinforcement learning (DRL) techniques to train an agent capable of achieving human-level performance playing the Atari video game [14]. DRL-based methods have also been applied to autonomous driving tasks [15,16].

The success of the above work in applying DRL to different decision-making problems has led us to apply DRL methods to the multi-robot autonomous exploration problem. In this paper, we propose applying the multi-agent deep reinforcement learning (MADRL) [17] approach to the multi-robot autonomous exploration task and employing the Transformer model [18] as part of the feature extractor. The main contributions of this paper are as follows:

- A novel DRL framework is introduced to address the challenge of multi-robot autonomous exploration.
- We propose a distributed decision network architecture based on the Transformer model.

## 2. Related Work

### 2.1. Multi-Robot Exploration

The frontier position is an essential concept in robotic exploration tasks, which refers to the position of the boundary between a known obstacle-free region and an unknown region during exploration. The nearest frontier method [9] is the simplest multi-robot autonomous exploration method. It continuously selects the closest frontier location to each robot as a target point for exploration. This simple strategy leads to repetitive exploration and inefficiency.

Utility-based approaches select exploration target points by manually constructing utility functions. Butzke et al. [10] and Burgard et al. [19] constructed utility functions from the information gain and the distance traveled by the robot. Matignon et al. [20] included information about robot interaction in creating the utility function to reduce communication losses between robots. Stachniss et al. [21] and Wang et al. [22] considered semantic and structural information about the environment, thus guiding the robot to be able to decentralize to different parts of the environment for exploration. In addition, Colares et al. [23] similarly achieved the same goal of spreading out the team of robots by considering the distance information between them. Utility-based approaches can enhance exploration efficiency, but when the number of elements evaluated increases, resulting in a more intricate utility function, it may become unsolvable owing to dimensional catastrophe.

In [8], a planning-based approach using a Decentralized Markov Decision Process (Dec-MDP) is presented and solved using online value iteration. When a robot teammate is out of communication range, the probability that the teammate is in a specific state is calculated using the known state obtained from the last communication and the elapsed time duration. In this way, it compensates for communication breakdowns and avoids the repeated exploration of areas that teammates have explored. In addition to the above classical approaches, some learning-based approaches have recently been proposed [24,25].

### 2.2. Deep Reinforcement Learning

DRL [11] was proposed to solve the problem of reinforcement learning (RL) [26] being unable to be used in high-dimensional spaces. Deep Q-Learning [27] was introduced as a solution to address the issue of Q-Learning’s excessively large Q-tables [28] while dealing with issues that have a high number of dimensions. Lillicrap et al. introduced the Deep Deterministic Policy Gradient (DDPG) technique [29] for application in systems with continuous action spaces. Haarnoja et al. proposed the Soft Actor–Critic (SAC) method [30], which maximizes the entropy of the action probability distribution while maximizing the expected reward to improve the efficiency of an agent’s exploration in the action space.

Most approaches in multi-agent reinforcement learning are founded on the Centered Training and Decentralized Execution (CTDE) framework [17]. MADRL approaches can be categorized into value-decomposition-based and actor–critic-based. Sunehag et al. introduced the Value-Decomposition Network (VDN) approach, which employs a centralized training process with a global value function [31]. Each agent uses its local value function for decision-making during distributed execution, and the global value function is the sum of all local value functions. Rashid et al. proposed the QMIX method, which changed the global value function to a nonlinear combination of individual local value functions based on the VDN [32]. Multi-Agent Deep Deterministic Policy Gradient (MADDPG) [33] and Multi-Agent Proximal Policy Optimization (MAPPO) [34] are two commonly used actor–critic-based multi-agent deep reinforcement learning methods. MADDPG is an offline method, while MAPPO is an online method.

## 3. Problem Statement

A multi-robot collaborative autonomous exploration task involves a group of robots exploring an unknown environment and building a map. Throughout the exploration procedure, each robot utilizes its sensors, such as LiDAR, to scan the surroundings and create its localized map. The robots then consistently transmit the local maps to the server. The decentralized execution of the exploration strategy enables each robot to choose a target location. At the same time, the path planning algorithm directs the robot towards the target location, allowing for the exploration of the surrounding environment during movement. When the separation between two robots is smaller than the communication range $d_{c}$, they can share their respective local maps, positions, and target locations. Throughout the exploration procedure, the server integrates the individual maps generated by each robot into the overall map in real time. Once the global map is fully assembled, the server issues an end command to each robot, signifying the completion of the exploration task.

The objective of the autonomous exploration strategy is to minimize the duration of the exploration process. Therefore, the objective function is defined as follows:

(1) $$\min\left[\sum_{i=1}^{T}t_{i}\right],$$

where *T* is the number of time steps in the exploration process, and $t_{i}$ is the duration of the ith time step.

**Figure 1:**
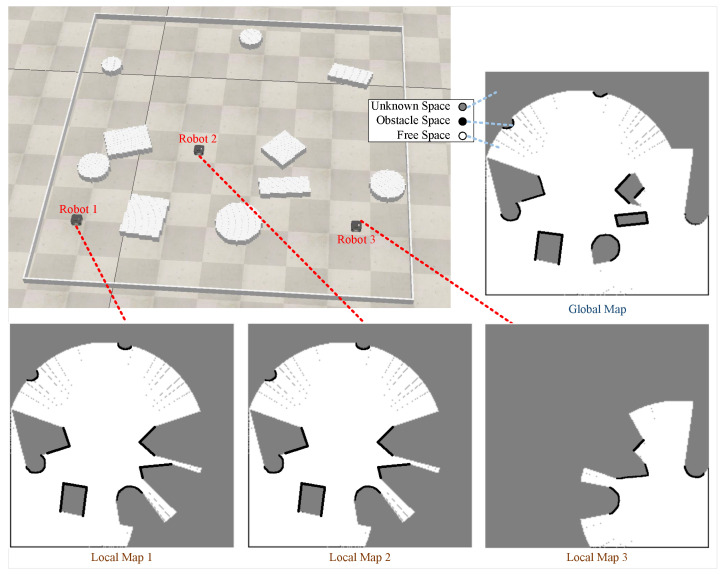
An example of the initial state of an exploration process.

Figure 1 depicts the initial state of an exploration process. The figure in the upper left corner shows three robots in an unknown environment. The local maps are the maps stored on the robots, and the global map is stitched together from all the local maps and stored on the server. The gray area on the map is the unknown space, the black area is the obstacle space, and the white area is the free space. Since LiDAR can only scan the edges of the obstacles, the obstacle space consists of these edges. We have bolded the edges of the obstacles for ease of viewing. Three robots are situated in an unknown environment, and each robot generates local maps by scanning their immediate surroundings. The local map of Robot 1 is identical to that of Robot 2. This occurs because the distance between these two robots is within the range of communication, allowing for the exchange of local maps. The global map is formed by combining all the individual local maps.

## 4. Methodology

This section proposes the novel TBDE-Net architecture to address the decentralized multi-robot autonomous exploration problem.

### 4.1. Model for Decentralized Multi-Robot Autonomous Exploration

We model the decentralized multi-robot autonomous exploration problem as a Decentralized Partially Observable Markov Decision Process (Dec-POMDP) [35]. A Dec-POMDP is defined by the tuple $\langle I,S,A,P,R,O,O,h\rangle$. In the tuple, *I* is the set of robots, *S* is the set of states, and A is the finite set of joint actions. $P\left(s^{\prime }|s,a\right)$ is the state transfer probability, which denotes the probability that, in state *s*, the robot team takes the joint action *a* and then transfers to state $s^{\prime }$, where $s,s^{\prime }\in S$, a∈A. $R\left(s,a,s^{\prime }\right)$ is a reward function that represents the reward acquired by the robot team in state *s* after performing the joint action *a* and then transferring to state $s^{\prime }$, where $s,s^{\prime }\in S$ and a∈A. O is the finite set of joint observations. $O\left(s^{\prime },z\right)$ is the observation function that gives the probability of obtaining the joint observation *z* when the state of the environment is at $s^{\prime }$, where $s^{\prime }\in S$ and z∈O. The goal is to find a set of distributed policies $\Psi ={\left\{\pi_{i}\right\}}_{i\in I}$ that can enable the team of robots to maximize the expected team rewards over the horizon *h* of an episode:

(2) $$\Psi^{*}=\arg\max_{\Psi }E\left[\sum_{t=0}^{h-1}\gamma^{t}R\left(s_{t},a_{t},s_{t+1}\right)|s_{0},\Psi \right],$$

where $\gamma \in \left(0,1\right]$ is a discount factor for future rewards, $s_{0}$ is the initial state.

### 4.2. Deep Reinforcement Learning

In this subsection, we introduce the innovative Transformer-Based Decentralized Exploration Network (TBDE-Net) framework. We refine our suggested network using the MADDPG MADRL technique [33]. The goal is to develop decentralized exploration methods.

**Figure 2:**
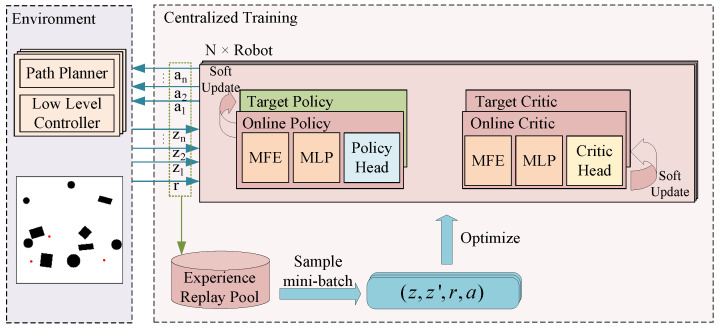
Structural diagram of the methodology of centralized training.

Figure 2 illustrates the framework structure of centralized training. The technique of DRL involves the selection of a goal point (action) to interact with the environment. A path planning algorithm and a low-level controller direct the robot toward the goal point. The robot scans the environment while moving, allowing for exploration. Simultaneously, the environment transmits the observations acquired by each robot to the centralized training framework. The centralized training framework records the actions, observations, and rewards of each encounter with the environment in the experience replay pool and samples mini-batches for training. In the figure, MFE denotes the Transformer encoder-based map feature extractor, and MLP is the multilayer perceptron. *z* is the joint observation information, *r* is the reward value, and *a* is the joint action. Every robot was equipped with a path planner and a low-level controller within the environment. The obstacles in the environments involved in this paper were static. During training, the A* path planning algorithm [36] (a path planning algorithm for environments of static obstacles) was used, and the kinematics and dynamics model of the robots, as well as the collisions between the robots, were not taken into account to increase the training speed. It was assumed that the robots could rigidly follow the programmed pathways. The A* path planning algorithm was utilized during simulation tests to plan the global path. In contrast, the Timed-Elastic-Band (TEB) local path planner [37] was used to plan the local path and provide control signals to direct the robot’s movement.

During centralized training, every robot was equipped with four networks: the online policy network, the target policy network, the online critic network, and the target critic network. The target and online policy networks had identical structures, differing only in their parameters. Similarly, the target and online critic networks also possessed the same structure, with parameter variations. The policy network comprised a Transformer-based map feature extractor, a multilayer perceptron, and a policy head. The critic network comprised a Transformer-based map feature extractor, a multilayer perceptron, and a critic head. The map feature extraction network of the policy network differed slightly from the map feature extraction network of the criterion network. This was because the input of the criterion network included an additional set of action information compared to the input of the policy network, resulting in a larger input dimension. Each robot’s observation included a partial map stored internally, as well as other robots’ positions and target positions within its communication range.

**Figure 3:**
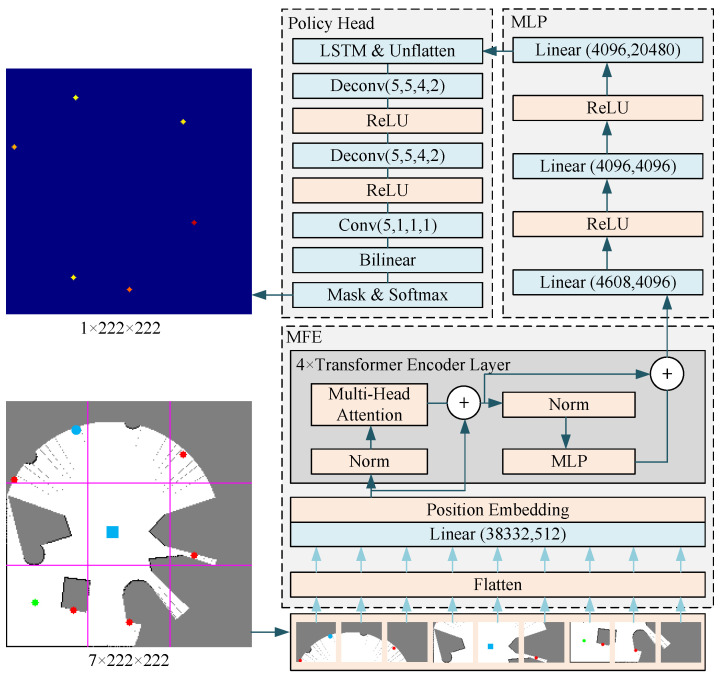
Policy net structure.

Figure 3 shows the network structure of the policy net. Position Embedding refers to sinusoidal position embedding, while Norm refers to layer normalization. Linear refers to a layer that performs linear transformations. The values in the parenthesis indicate the dimensions of the input and output, respectively. Unflatten refers to rearranging data from a one-dimensional format into a three-dimensional one with dimensions of 5 × 64 × 64. Deconv refers to the operation of transposed convolution. The numbers enclosed in parentheses represent the dimensions of the input features, output features, kernel size, and stride size. Conv refers to a convolutional layer. The values in parentheses hold the same importance as those in transposed convolution. Bilinear refers to bilinear resampling for a final image size of 222 × 222 pixels. A mask selectively filters the prospective target’s location within the action space. The map consists of different sections: the white area represents free space, the gray area represents unknown space, and the black area represents obstacle space. The green dots indicate the robot’s current position, and the red dots represent potential target positions that might be chosen (action space). The blue dots on the map represent other robots within the communication range, while the blue squares represent their target positions. This network’s input data size is 7 × 222 × 222. These seven data layers correspond to the unknown space, the free space, the obstacle space, the robot’s position, the action space, the position of other robots within the communication range, and the target points of other robots within the communication range. The input data are evenly divided into nine parts according to the line drawn in the figure. MFE unfolds these nine parts into one-dimensional data and performs position embedding after mapping them through a linear layer. In this paper, the position embedding method was sinusoidal position embedding. The data are fed into the MLP after passing through the Transformer encoder layer, which consists of 4 layers. The Transformer encoder layer consisting of 4 layers is input into the MLP. The model dimension of the Transformer encoder is 512, and the number of heads in the multi-head attention layer is 8. In the policy head, a Long Short-Term Memory (LSTM) network first allows the robot to memorize some previously communicated information about other robots, such as implicit expressions of other robots’ intentions. After that, the one-dimensional data are reconstructed into five layers of 64 × 64 two-dimensional data, which are transformed into one layer of 222 × 222 two-dimensional data through a series of transposed convolutional layers and resampling. After a mask operation, we filter out the data in the action space through a softmax layer to obtain the action probability distribution.

**Figure 4:**
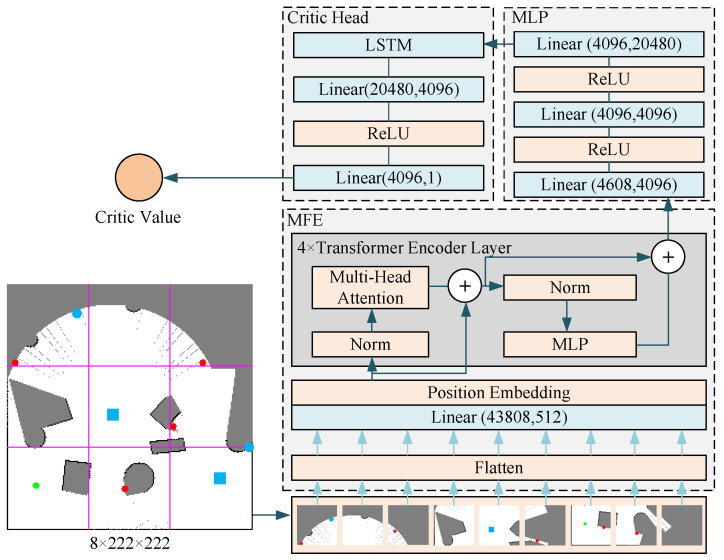
Critic net structure.

Figure 4 illustrates the structure of the critic net. The critic net and policy net differ only in the MFE and the critic head. The input data of the MFE for the critic net are global observations instead of local observations in the policy net and also have one more layer of action probability distribution information than the input data of the policy net. The critic head is an MLP that ultimately outputs an element used to evaluate the goodness of the action. The input data of the critic net consist of 8 layers of data: unknown space, free space, obstacle space, the robot’s own position, frontier positions, other robots’ positions, other robots’ target positions, and action probability distribution information. The MFE part differs from the policy net only in the input dimension, and the MLP part is the same as that of the policy net. In the critic head, it is a multilayer perceptual machine that goes through multiple linear layers and activation functions to finally output an evaluation of the probability distribution of the action.

The action space is the range of actions a reinforcement learning method can perform. In this paper, the action space referred to the set of candidate locations for exploration. In this paper, we used all the frontier points in the local map of each robot as the action space and used a machine vision-based approach for the frontier point extraction:

1. The local map was represented by a grayscale image with a depth of 8 bits, where the pixel values of the free space, obstacle space, and unknown space were 200, 100, and 0. Edge extraction was carried out by the Canny operator with high and low thresholds of 650 and 600, respectively, and the extracted edges made up the set of all the frontier pixels.
2. All connected domains were found for the pixels in the set, and the center of each connected domain was calculated, thus clustering the connected frontier pixels.
3. For each center position, the nearest pixel that belonged to the free space was calculated as a frontier point in the action space.

The performance of the strategy derived from the final training, as well as the training speed, is influenced by the design of the reward function. In this paper, all robots shared a reward function. The reward function was defined as follows:

(3) $$r_{explore}=\left(U_{t}\cap E_{t}\right),\;\mathrm{each}\;\mathrm{step},$$

(4) $$r_{repeat}=-\left(C_{t}\cap E_{t}\right),\;\mathrm{each}\;\mathrm{step},$$

(5) $$r_{time}=-T_{t},\;\mathrm{each}\;\mathrm{step},$$

(6) $$r_{step}=-1,\;\mathrm{each}\;\mathrm{step},$$

(7) $$r_{complete}=+100,\;\mathrm{complete}.$$

The reward function was the sum of five terms: the new exploration area, the repeated exploration area, exploration time, the decision count penalty, and the completed exploration reward. In the equation, $U_{t}$ is the unknown region, $E_{t}$ is the region explored in this step, $C_{t}$ is the region explored before this step, and $T_{t}$ is the time elapsed. In our case, the unit of the subitem relating to the area was square meters, and the unit of the subitem relating to time was seconds. When exploring, robot teams were rewarded for exploring unknown areas and penalized for repeatedly exploring an area, thus reducing the need for robots to repeat scans of areas they had already explored. A fixed slight penalty was imposed on each decision. This was to prevent the neural network from constantly choosing the nearest position to explore in the case of multiple frontier positions with similar locations, which would cause the robot to engage in frequent decision-making processes and waste time. When a team of robots finished exploring an unknown environment, it received a fixed reward.

The experience replay pool stores the interactions between the robot team and the environment to optimize the neural network. The replay buffer for each contact with the environment can be represented as a vector, $b=\langle Z_{t+1},A_{t},R_{t}\rangle$, where $Z_{t}=\left\{z_{0},z_{1},\ldots ,z_{t}\right\}$, $A_{t}=\left\{a_{0},a_{1},\ldots ,a_{t}\right\}$, and $R_{t}=\left\{r_{0},r_{1},\ldots ,r_{t}\right\}$ denote the joint observation, joint action, and reward from the start of the episode to step *t*, respectively. In contrast to the typical experience replay pool in MADDPG, we systematically stored all collective observations, actions, and rewards from the start of the episodes until the t-step. The existence of LSTM modules in our network necessitated the use of entire sequences in order to create memory information.

### 4.3. Exploration Process

The exploration process consisted of a communication process, a navigation process, a map-building process, and a decision-making process. For each robot in the robot team, the local map was exchanged if a teammate was within its communication range during the exploration process. The navigation process planned a feasible path through the robot’s current position and the goal point’s location, given by the decision process. As the robot traveled along the path, the map-building process constructed a map of the unknown environment based on the environmental information scanned by the robot. When the robot reached the goal point location, the decision-making process used TBDE-Net to select a point among the frontier positions as a new goal point to explore. This process was looped continuously until the environment was fully explored. In other words, the communication, navigation, and map-building processes ran parallel throughout the exploration process, while the decision-making process ran periodically.

In the decision-making process, the robot extracted all the frontier positions through the local map and obtained the probability distribution of selecting each frontier position through the policy network. Among them, the input data of the policy network included the local map, the frontier positions in the local map, the robot’s position, and the position and target position of the teammate at the last communication with each teammate. During training, a frontier position was randomly selected as the target point through the probability distribution returned by the strategy network. In contrast, the frontier position with the largest probability was directly selected as the target point during testing.

During training, the A* path planning algorithm was used, and it was assumed that the robot could travel strictly according to the planned path without considering the robot’s dynamics model to save training time. During testing, the global path was planned using the A* path planning algorithm, and the velocity signal was generated using the TEB local path planner, and a low-level controller guided the robot motion. The Hector mapping algorithm was used in this paper for the map-building process.

## 5. Training

This section provides a comprehensive overview of the training process and illustrates how the model’s performance evolves throughout the training.

### 5.1. Training Environment Generation

**Figure 5:**
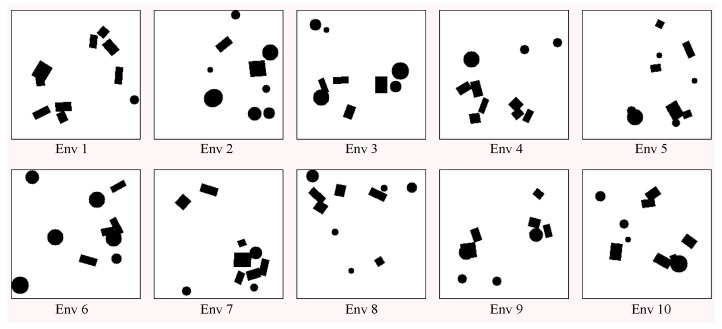
Randomly generated training environments.

The training environments were generated randomly, following the following process. A circular boundary with a width of 1 pixel was positioned along the perimeter within an empty area measuring 222 × 222 pixels. The resolution of the generated environment was 0.1 m per pixel. Subsequently, ten obstacles were distributed evenly and haphazardly over the area, with their shapes being randomly chosen among circles and squares. The radius of the circular obstacles followed a uniform distribution U(5,15). The side sizes of square obstacles followed a uniform distribution U(10, 30) and were randomly rotated. These obstacles could overlap. The resolution of the environment was 0.1 m per pixel. Using the abovementioned method, we randomly generated ten environments for training, as shown in Figure 5. Each environment consisted of ten randomly generated obstacles that could overlap each other.

### 5.2. Training Process

We trained the neural network using an Intel i5 12400f CPU and an NVIDIA RTX3060 GPU. We conducted training sessions with teams of robots consisting of two, three, and four robots, respectively, within training environments. During training, we used the A* path planning algorithm and assumed that the robot could move strictly according to the planned paths. The robot’s kinematics and dynamics models were not taken into account to speed up the training. The relevant parameters for training are shown in Table 1.

**Figure 6:**
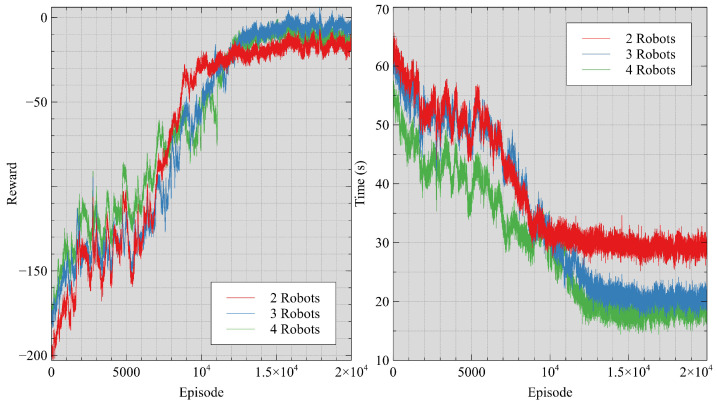
Policy performance varied with the number of training episodes.

The change in the policy’s performance with the training process is shown in Figure 6. The left graph illustrates the variation in average rewards obtained by robot teams of varying sizes during their training process. The right graph shows the average time it takes for robot teams of different sizes to explore unknown environments through the training progresses. The training for each robotics team size required an average of 18 h. The network could be trained to reach convergence within 20,000 episodes.

## 6. Experiment

Within this section, we formulated two sets of experiments: a comparative experiment and a test to assess generalization capabilities. In our comparative tests, we assessed the effectiveness of our proposed strategy by comparing it to traditional and learning-based methods. We constructed a scenario within the Vrep simulation environment during the generalization ability test. Next, we evaluated the robot’s kinematics and dynamics model to determine if our proposed method could be successfully applied in a nearly realistic setting.

### 6.1. Comparative Experiment

We compared TBDE-Net with the following methods:

- Nearest frontier [9]: each robot continuously selects the closest frontier location as its target point until the environment is fully explored.
- Next-Best-View [23]: each robot selects a target point to explore by evaluating the distance from the candidate point to itself and the information gain available at the target point.
- Planning-based [8]: each robot uses Dec-MDP for online planning and reduces repetitive exploration of the environment by predicting the state of other robots from locally observed information.
- DME-DRL [25]: the distributed multi-robot exploration algorithm based on deep reinforcement learning (DME-DRL) explores by selecting the nearest frontier position in a particular direction as a target point.

Like our proposed method, the DME-DRL method is also based on deep reinforcement learning. It is based on the convolutional neural network, while our method is based on the Transformer model. On the other hand, the output of the DME-DRL method is directional information, and it selects the nearest frontier point in the output direction as the target point, while our method directly selects among all the frontier points with a larger action space.

In the comparison experiment, we randomly generated 100 environments for the comparison experiment. In each environment, teams of robots of different sizes completed ten separate explorations using various methods at communication distances of 5 and 8 m, respectively. At the beginning of each exploration, the position of each robot was randomized. Throughout the exploration, the velocity of each robot remained constant at 1 m per second. Meanwhile, path planning was performed using the A* algorithm, and the robot was assumed to follow the planned path strictly.

**Figure 7:**
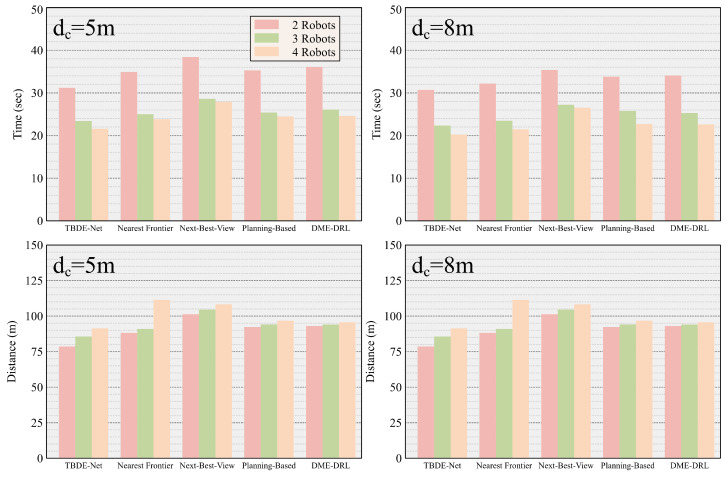
The average time spent and the average total distance traveled to complete the exploration by different methods.

Figure 7 shows the time and distance spent by robot teams of different sizes using different methods to explore an unknown environment at two different communication distances. The TBDE-Net-based method outperformed other methods in both time and distance cost. Table 2 shows the time and distance traveled by each method to complete the exploration of the unknown environment. The experimental results show that the TBDE-Net-based, planning-based, and DME-DRL methods were insensitive to the variation in the communication distance. This is because both the TBDE-Net-based method and the DME-DRL method learn an implicit representation of the teammate’s state when communication is interrupted, and the planning-based method extrapolates the teammate’s current state from the state of the teammate before communication is interrupted.

**Figure 8:**
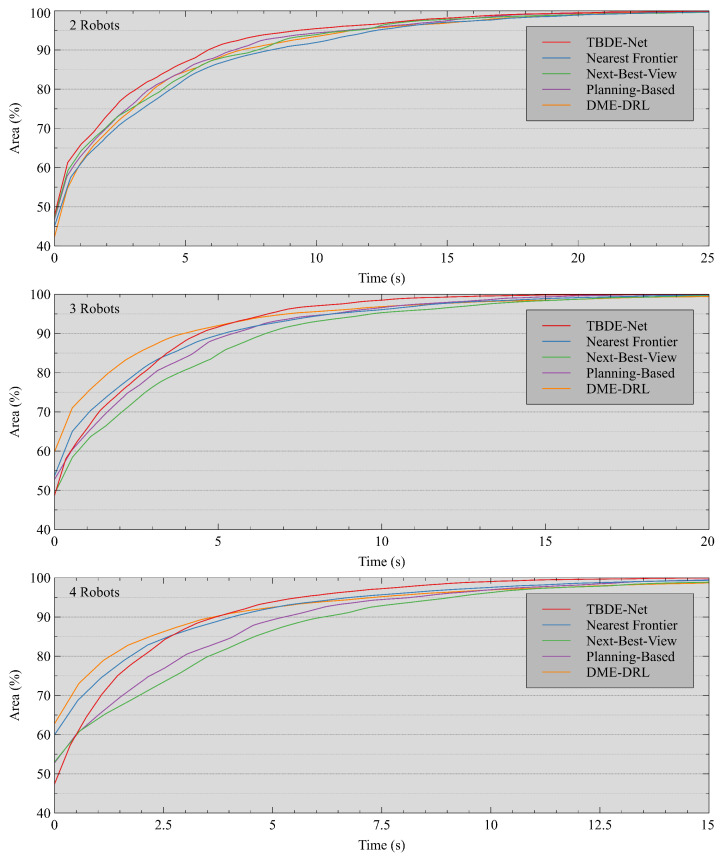
Exploring progress–time curves. The three figures correspond to robot teams of sizes 2, 3, and 4, respectively.

Figure 8 depicts the relationship between the average exploration progress and exploration time for various methods. In each graph, the horizontal axis represents time, while the vertical axis represents the average exploration progress (the area of the explored region as a proportion of the area of the free space of the environment). When the size of the robot team was two, our proposed method led in exploration progress at almost all moments. At robot team sizes three and four, our proposed method did not explore as fast as the DME-DRL-based and nearest frontier-based methods in the pre-exploration phase but achieved a reversal in the post-exploration phase. This is because both the nearest frontier and the DME-DRL-based methods always select the nearest frontier position or the nearest frontier position in a specific direction. They focus too much on localized exploration and miss out on the big picture, generating many duplicated explorations in the later stages.

### 6.2. Generalization Capability Test

We built a simulation environment in Vrep and deployed our method in the Robot Operating System (ROS) framework to test the generalization ability of our proposed method.

**Figure 9:**
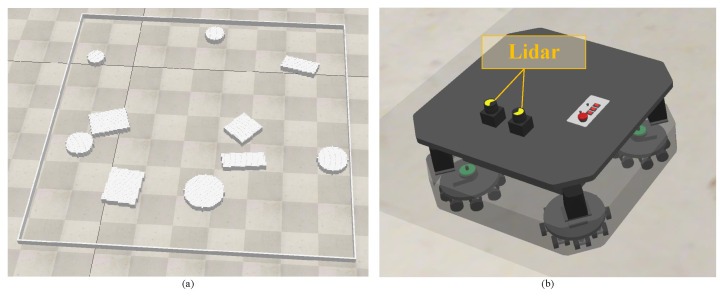
Test environment and the robot. (**a**) The environment, (**b**) the robot.

Figure 9 shows the test environment we built in Vrep. The size of the test area was 22.2 m by 22.2 m. Each test robot included an omnidirectional motion mechanism and was equipped with two Hokuyo URG 04LX LiDARs to scan the surrounding area in a 360-degree manner. The robot’s maximum velocity was 1 m per second.

In the test, the robots used the move base package provided in ROS with the A* global path planner and the TEB local path planner to plan the path and control the robot’s motion. The robot used the Hector mapping algorithm to construct local maps from the data returned by the LiDAR. During the exploration process, TBDE-Net was used to select the target points. The local maps of each robot were spliced into the global map at the map splicing node, and the exploration was considered to be completed when there was no frontier unknown in the global map. We conducted ten explorations in a simulated environment, utilizing teams of robots consisting of two, three, and four robots, respectively. The beginning placements of the robots were randomized for each exploration.

**Figure 10:**
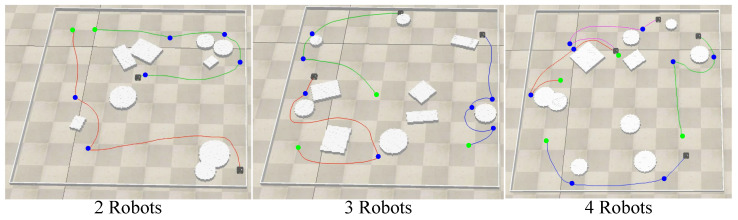
Path of different-size robotics teams to complete the exploration.

Figure 10 shows the paths traveled by the robots as three different-size robot teams completed their exploration of random unknown environments. The curves in the subfigures represent the paths traveled by the robots during exploration. The green points are the initial points, and the blue are the target points the robots passed during exploration. Table 3 demonstrates the test results with the nearest frontier method as a baseline. The experimental results show that TBDE-Net could explore unknown environments in a near-realistic simulation environment that considered the robot dynamics model, including its inertia and acceleration and deceleration processes.

## 7. Conclusions

This article proposed a decision network for solving multi-robot collaborative exploration problems based on the Transformer model. We used the MADDPG multi-agent reinforcement learning framework with our designed reward function to train our proposed network. Experiments demonstrated that our proposed method had advantages over the current mainstream traditional and learning-based methods in terms of exploration speed and distance loss. However, some additional work needs to be carried out. Since the input dimension of the neural network is fixed, it may not be applicable when dealing with unknown environments of different sizes. Also, the neural network must be retrained when the robot team size changes, which is more troublesome when used in scenarios where the robot team size is not fixed. In future research, we will consider that the policy networks of all robots share the same set of parameters to cope with variations in the size of the robot team and try to use networks of feature pyramid structures to adapt to environments of different sizes.

## Figures and Tables

**Table 1:** Relevant parameters for training.

Parameter Name	Value
Optimizer	Adam
Initial learn rate	0.001
Batch size	64
Replay buffer size	10,000
Communication range	5 m

**Table 2:** The time and distance traveled to complete the exploration by different methods.

Robotics Team Size	Method	Time (s)	Distance (m)
2	TBDE-Net	31.2	78.7
	Nearest frontier	34.6	88.4
	Next-Best-View	38.2	101.8
	Planning-based	35.9	92.2
	DME-DRL	36.0	93.7
3	TBDE-Net	23.0	85.3
	Nearest frontier	25.6	91.5
	Next-Best-View	28.8	104.7
	Planning-based	25.8	94.9
	DME-DRL	26.0	94.4
4	TBDE-Net	21.3	91.9
	Nearest frontier	23.6	111.9
	Next-Best-View	27.4	108.1
	Planning-based	24.6	96.4
	DME-DRL	24.4	95.2

**Table 3:** Generalization capability test result.

Robot Team Size	Method	Time (s)	Distance (m)
2	TBDE-Net	31.2	78.7
	Nearest frontier	39.1	89.9
3	TBDE-Net	25.6	99.3
	Nearest frontier	26.3	103.7
4	TBDE-Net	23.5	107.1
	Nearest frontier	27.2	127.3

## Data Availability

Data are contained within the article.

## Abbreviations

The following abbreviations are used in this manuscript:

DRL	Deep reinforcement learning
MADRL	Multi-agent deep reinforcement learning
RL	Reinforcement learning
DDPG	Deep Deterministic Policy Gradient
SAC	Soft Actor–Critic
CTDE	Centered Training and Decentralized Execution
VDN	Value-Decomposition Network
MADDPG	Multi-Agent Deep Deterministic Policy Gradient
MAPPO	Multi-Agent Proximal Policy Optimization
Dec-POMDP	Decentralized Partially Observable Markov Decision Process
TBDE-Net	Transformer-Based Decentralized Exploration Network
TEB	Timed-Elastic-Band
LSTM	Long Short-Term Memory
Dec-MDP	Decentralized Markov Decision Process
DME-DRL	Distributed multi-robot exploration algorithm based on deep reinforcement learning
ROS	Robot Operating System
